# Protective Vaccination against Blood-Stage Malaria of *Plasmodium chabaudi*: Differential Gene Expression in the Liver of Balb/c Mice toward the End of Crisis Phase

**DOI:** 10.3389/fmicb.2016.01087

**Published:** 2016-07-14

**Authors:** Saleh A. Al-Quraishy, Mohamed A. Dkhil, Abdel-Azeem A. Abdel-Baki, Denis Delic, Frank Wunderlich

**Affiliations:** ^1^Department of Zoology, College of Science, King Saud UniversityRiyadh, Saudi Arabia; ^2^Department of Zoology and Entomology, Faculty of Science, Helwan UniversityCairo, Egypt; ^3^Department of Zoology, Faculty of Science, Beni-Suef UniversityBeni-Suef, Egypt; ^4^Boehringer-Ingelheim PharmaBiberach, Germany; ^5^Department of Biology, Heinrich-Heine-UniversityDuesseldorf, Germany

**Keywords:** blood-stage malaria, *Plasmodium chabaudi*, protective vaccination, gene expression, lincRNA, hepatic erythropoiesis, liver regeneration

## Abstract

Protective vaccination induces self-healing of otherwise fatal blood-stage malaria of *Plasmodium chabaudi* in female Balb/c mice. To trace processes critically involved in self-healing, the liver, an effector against blood-stage malaria, is analyzed for possible changes of its transcriptome in vaccination-protected in comparison to non-protected mice toward the end of the crisis phase. Gene expression microarray analyses reveal that vaccination does not affect constitutive expression of mRNA and lincRNA. However, malaria induces significant (*p* < 0.01) differences in hepatic gene and lincRNA expression in vaccination-protected vs. non-vaccinated mice toward the end of crisis phase. In vaccination-protected mice, infections induce up-regulations of 276 genes and 40 lincRNAs and down-regulations of 200 genes and 43 lincRNAs, respectively, by >3-fold as compared to the corresponding constitutive expressions. Massive up-regulations, partly by >100-fold, are found for genes as *RhD, Add2, Ank1, Ermap*, and *Slc4a*, which encode proteins of erythrocytic surface membranes, and as *Gata1 and Gfi1b*, which encode transcription factors involved in erythrocytic development. Also, *Cldn13* previously predicted to be expressed on erythroblast surfaces is up-regulated by >200-fold, though claudins are known as main constituents of tight junctions acting as paracellular barriers between epithelial cells. Other genes are up-regulated by <100- and >10-fold, which can be subgrouped in genes encoding proteins known to be involved in mitosis, in cell cycle regulation, and in DNA repair. Our data suggest that protective vaccination enables the liver to respond to *P. chabaudi* infections with accelerated regeneration and extramedullary erythropoiesis during crisis, which contributes to survival of otherwise lethal blood-stage malaria.

## Introduction

Malaria has caused worldwide about 214 million new cases and about 438,000 deaths in 2015 (WHO, 2015). The liver plays an important role in malaria. It is the organ of intra-hepatocyte multiplication of the pre-erythrocytic stages of the malaria-causing agent, parasitic protozoans of the genus *Plasmodium* (Bertolino and Bowen, 2015; Frevert and Krzych, 2015; Frevert et al., 2015). Moreover, the liver responds to the morbidity- and mortality-causing blood-stages of the parasites. The liver is pathologically changed and even massively injured in patients suffering from blood-stage malaria (Ananad et al., 1992; Kochar et al., 2003; Nautyal et al., 2005; Rupani and Amarapurkar, 2009). Also, the liver with its inherent immune system even functions as an effector against blood-stage malaria (Dockrell et al., 1980; Abo and Sekikawa, 2002; Wunderlich et al., 2005, 2014). The latter view is supported by studies performed with animal models, as e.g., *Plasmodium chabaudi* in mice. This model shares several characteristics with *P. falciparum*, the most dangerous malaria species for humans (Longley et al., 2011; Stephens et al., 2012). Mice are either susceptible or resistant to *P. chabaudi* malaria, i.e., blood-stage infections take either a lethal or a self-healing outcome. This outcome is under complex control involving genes of the mouse-MHC, the *H-2* complex, and genes of the non-*H-2* background as well as environmental factors (Wunderlich et al., 1988b, 2014). Both lethal and self-healing infections take a similar course of peripheral parasitemia during the pre-crisis phase. For instance, when mice are challenged with 10^6^ *P. chabaudi*-infected erythrocytes, precrisis culminates at peak parasitemia of about 40–50% on approximately day 8 *p.i.* The following crisis phase, characterized by dramatically declining peripheral parasitemia to about 2–1% within about 3–4 days, is apparently critical for the outcome of disease. Indeed, all susceptible mice succumb during crisis, whereas resistant mice will survive and generate long-lasting immune mechanisms against homologous re-challenge (Wunderlich and Helwig, 1987).

An effective anti-malaria vaccine for humans is currently not yet available. However, there are promising candidate vaccines in progress (Halbroth and Draper, 2015; Hoffman et al., 2015; Miura, 2016). To date, only RTS,S/AS01 vaccine has completed a phase 3 evaluation (White et al., 2015). Although RTS,S/AS01 received a positive regulatory assessment by the European Medicine Agency for its active immunization of children aged 6 weeks to 17 months against malaria it was not recommended by the WHO (Birkett, 2016; Gosling and von Seidlein, 2016). Moreover, the protective mechanisms of the host defense, which have to be activated by a vaccine, appear to be not yet fully understood despite enormous advances in our knowledge during the past 35 years. At least, however, those protective mechanisms directed against blood-stage malaria can be also conveniently investigated in the *P. chabaudi* model. Indeed, there has been previously developed a vaccination procedure which raises survival of malaria-susceptible mice from 0 to over 80% (Wunderlich et al., 1988a; Krücken et al., 2009). This vaccination, using non-infectious surface membranes isolated from *P. chabaudi*-infected erythrocytes, does not induce sterile immunity, but converts lethal blood-stage infections to take a self-healing course. Moreover, there is information available, though still little, that protective vaccination also affects the liver (Krücken et al., 2009; Wunderlich et al., 2014). This becomes evident especially during the crisis phase, when vaccination leads to a dramatic increase in the hepatic capacity to trap injected particles (Krücken et al., 2009).

The processes associated with protective vaccination in the liver during crisis are only poorly investigated to date. To trace these processes, it would be initially meaningful to identify those genes whose expression is changed in the liver of vaccination-induced self-healing infections toward the end of the crisis phase. This would in turn allow deductions for processes possibly involved in the liver to mediate protection against blood-stage malaria. We have therefore decided to analyze the effect of protective vaccination on the transcriptome of the liver toward the end of the crisis phase in vaccination-induced self-healing infections of *P. chabaudi* in comparison to lethal infections in non-protected Balb/c mice using Agilent's gene expression microarrays.

## Materials and methods

### Mice

This study continues our previous work performed with female Balb/c mice (Krücken et al., 2009). It was carried out in strict accordance with the German law on animal protection. The keeping of mice as well as the experimental protocol of the study were officially approved by the State-controlled Committee on the Ethics of Animal Experiments of the State Nordrhein-Westfalen, Germany, and were regularly controlled, without being previously announced, by the local authorities. All efforts were made to minimize suffering. Female Balb/c mice were delivered at an age of 10–12 weeks from our central animal facilities at the University of Düsseldorf, where they were bred under specified pathogen-free conditions. During the experiments, the mice were housed in plastic cages, received a standard diet (Woehrlin, Bad Salzuflen, Germany) and water *ad libitum*.

### Blood-stage malaria

Blood-stage infections of *P. chabaudi* were routinely maintained in outbred mice under sterile conditions by weekly passages of infected blood. Since 1982, a non-clonal line of *P. chabaudi* is continuously used in our laboratory (Wunderlich et al., 1982) resembling to *P. chabaudi* as described previously (Wunderlich et al., 2005; Krücken et al., 2009). Balb/c mice were challenged *i.p*. with 10^6^ *P. chabaudi chabaudi* AS-infected erythrocytes. Parasitemia was evaluated in Giemsa-stained smears from tail blood. Erythrocytes were counted in a Neubauer chamber.

### Protective vaccination

Mice were vaccinated as detailed previously (Krücken et al., 2009). The vaccine contained host cell plasma membranes isolated in the form of ghosts from *P. chabaudi*-parasitized red blood cells as detailed previously (Wunderlich et al., 1985, 1987). These membranes were previously characterized to be non-infectious and to be associated with parasite-synthesized proteins (Wunderlich et al., 1988c,d) as it was also reported for surface membranes of *P. falciparum*-infected erythrocytes (Fontaine et al., 2012). Approximately 10^6^ ghosts were suspended in 100 μl Freund's complete adjuvant (FCA) and subcutaneously injected 3 and 1 week before infecting with *P. chabaudi*-parasitized erythrocytes. Controls received only FCA.

### RNA isolation

Livers were aseptically removed from sacrificed mice, rapidly frozen in liquid nitrogen and stored at −80°C until use. Frozen livers were grounded in a mortar under liquid nirogen and aliquots of each liver were used to isolate total RNA by the Trizol (Qiagen, Hilden, Germany) standard RNA extraction protocol. Trizol-extracted RNA was additionally cleaned up using the miRNeasy Kit (Qiagen). Integrity and quality of RNA was checked with the Agilent 2100 Bioanalyzer platform (Agilent Technologies). All RNA samples revealed RIN values between 8.7 and 9.1.

### Cy3-labeling of RNA

Equivalents of 100 ng from each RNA sample were used for the linear T7-based amplification step. To produce Cy3-labeled cRNA, the RNA samples were amplified and labeled with the Agilent Low Input Quick Amp Labeling Kit (Agilent Technologies) according to the manufacturer's instructions. Yields of cRNA and dye-incorporation were determined with the ND-1000 Spectrophotometer (NanoDrop Technologies). The incorporations were between 18 and 23 fmol Cy3/ng cRNA.

### Hybridization of agilent mouse whole genome oligo microarrays

Hybridization was performed with Agilent's 8 × 60 K oligo microarrays (design 028005), which contain 8 arrays per slide with each array displaying 39,430 Entrez Gene RNAs and 16,251 lincRNAs. Using the Agilent Gene Expression Hybridization Kit, hybridization was carried out as detailed in the Agilent processing protocol (Agilent technologies). In brief, 600 ng Cy3-labeled fragmented cRNA in hybridization buffer was hybridized overnight at 65°C to the microarrays using Agilent's recommended hybridization chamber and oven. Finally, the microarrays were washed with the Agilent Gene Expression Wash Buffer 1 for 1 min at room temperature, which was followed by washing with preheated Agilent Gene Expression Wash Buffer 2 at 37°C for 1 min.

### Scanning and data analysis

The Agilent's Microarray Scanner System (Agilent Technologies) was used to detect fluorescence signals on the hybridized microarrays. The microarray image files were read out and processed with the Agilent Feature Extraction Software. The latter determines feature intensities (including background substraction), rejects outliers and calculates statistical confidences. A heatmap was generated to visualize the expression levels of each mRNA and lincRNA (Spotfire, TIBCO Software Inc., Palo Alto, USA). The row and column dendrograms were clustered with the unweighted pair group method with arithmetic mean and Euclidean distance measure. Data are publicly available at the EMBL-EBI Array Express repository (Array Express accession number: E-MTAB-4791).

### Quantitative real-time PCR

Using High Capacity cDNA Reverse Transcription Kit (Life Technologies) and TaqMan mRNA assays (Life Technologies) we performed reverse transcription of mRNAs coding for the following proteins: RHD (assay ID: Mm00456910_m1), ERMAP (Mm_00469273_m1), ADD2 (Mm00478923_m1), ANK1 (Mm00482889_m1), PLK1 (Mm00440924_g1), CCNA2 (Mm00438063_m1), LIFR (Mm00442942_m1), and CD163 (Mm_00474091_m1). PCR reactions were performed with the TaqMan® gene expression master mix (Life Technologies) according manufacturer's protocol on a 7900HT real-time PCR System, as described previously (Al-Quraishy et al., 2014). All samples were run in duplicates and raw *ct* values were calculated using the SDS software v.2.4 and GAPDH was used for normalization. Fold change of expression was calculated with the comparative Ct method (2^−ΔΔct^) (Livak and Schmittgen, 2002). Data sets were analyzed for statistical significance using two-tailed unpaired heteroskedastic Student's *t*-test (^*^p < 0.01).

## Results

### Identification of differentially expressed genes in the liver of vaccination-protected vs. non-protected mice

**Figure 1:**
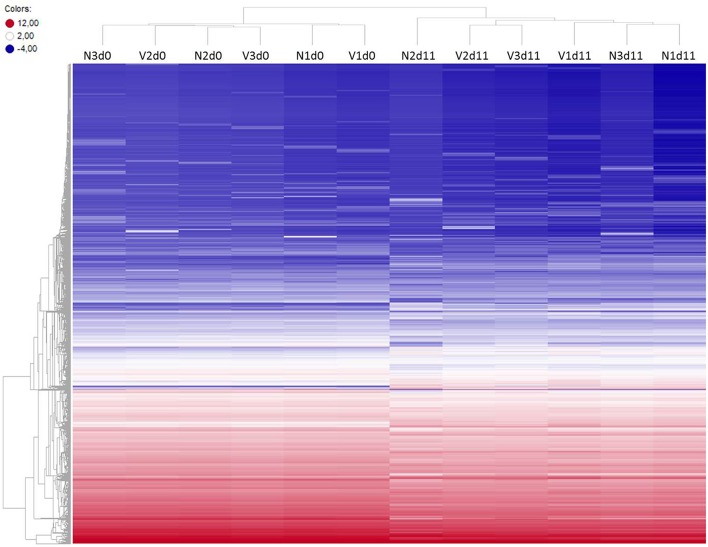
**Heatmap of global expression levels of mRNA and lincRNA in the liver of non-vaccinated (N) and vaccinated (V) mice on day 0 *p.i.* (Nd0, Vd0) and day 11 *p.i.* (Nd11, Vd11)**. Expression levels for each sample were hierarchically clustered. Log_2_ transformed expression levels range from −4 to 12 indicated by blue and red color, respectively.

To identify differentially expressed genes in the liver of vaccination-protected mice, in comparison with non-vaccinated malaria-susceptible mice, toward the end of the crisis phase of blood-stage infections of *P. chabaudi*, we have analyzed gene expression microarrays from livers individually prepared from three vaccination-protected mice on day 11 *p.i.* (Vd11) and from 3 vaccinated mice before infection on day 0 *p.i.* (Vd0) as well as from 3 non-vaccinated mice on day 0 *p.i.* (Nd0) and on day 11 *p.i.* (Nd11). The course of parasitemias of *P. chabaudi* infections in vaccinated and non-vaccinated mice has been previously determined under identical experimental conditions as used here (Krücken et al., 2009). The parasitemia was 0% at Vd0 and Nd0 and approximately 2% at Vd11 and Nd11, respectively. Then, we have stringently selected for those genes, which displayed more than 3-fold changed mRNA expression levels at a significance level of *p* < 0.01 at Vd11 and Nd11 relative to the corresponding constitutive mRNA expressions at Vd0 and Nd0, respectively. Figure 1 shows the heat maps of the expression profiles of all self-healing and lethal infections on day 0 *p.i.* and day 11 *p.i.* The profiles of Vd0 and Nd0 separately cluster from those of Vd11 and Nd11, respectively.

**Figure 2:**
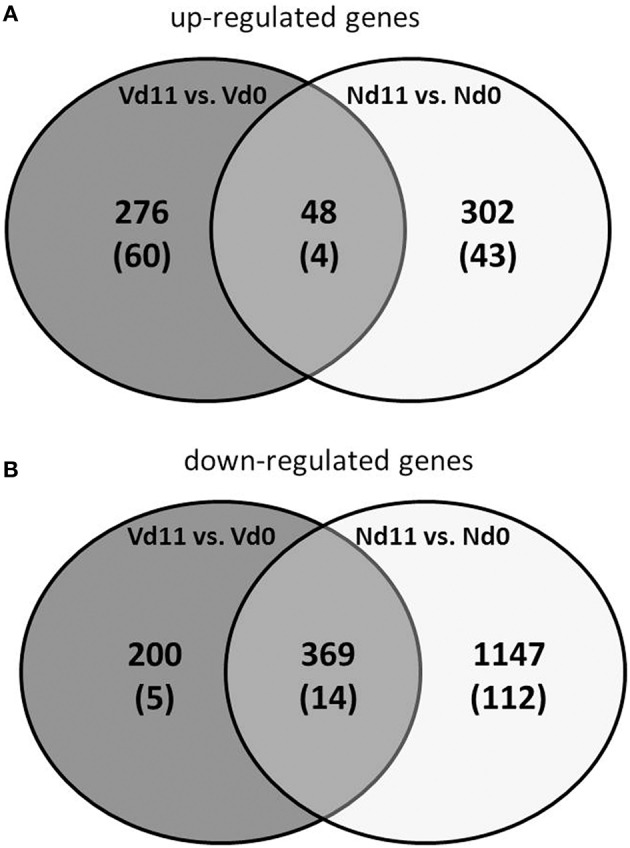
**Venn-diagram of the number of up-regulated (A) and down-regulated (B) genes expressed more than 3-fold (*p* < 0.01) in the liver of vaccinated mice infected with *P. chabaudi* toward the end of the crisis phase on day 11 *p.i.* (Vd11) in comparison to constitutive expressions day 0 *p.i.* (Vd0)**. Nd11 vs. Nd0 indicates number of those genes identified in the liver of non-vaccinated mice. Numbers in brackets indicate genes more than 10-fold changed.

Protective vaccination does not affect constitutive mRNA expression in the liver. However, the *P. chabaudi* infections induce significant changes in the expression of numerous hepatic genes, which differ between vaccination-protected and non-vaccinated Balb/c mice toward the end of the crisis phase of blood-stage infections of *P. chabaudi* on day 11 *p.i*. The Venn diagrams in Figure 2 summarize the number of identified genes: malaria induces 302 genes to be up- and 1,147 genes to be down-regulated by more than 3-fold at Nd11 in relation to their constitutive expressions at Nd0. The identity of these genes is provided in the Tables S1–S4. Tables S1, S2 list the genes whose expression is up- and down-regulated by more than 3-fold and less than 10-fold, respectively. In Tables S3, S4, those genes are summarized whose expression is more than 3-fold up and less than 10-fold down-regulated, respectively, in the liver of non-vaccinated mice.

Moreover, Figure 2 shows 48 and 369 genes exhibiting malaria-induced up- and down-regulated expressions, respectively, by more than 3-fold both at Nd11 and at Vd11 as compared with their constitutive expressions at Nd0 and Vd0. The Tables S5, S6 summarize those genes, whose expression is up- and down-regulated, respectively, by more than 3-fold and less than 10-fold in the liver of both vaccinated and non-vaccinated mice. Table 1 lists those 4 genes and 14 genes, respectively, whose expressions are significantly up- and down-regulated by more than 10-fold in both non-vaccinated susceptible mice and vaccination-protected mice (Figure 2). However, the differences between both groups are not larger than about 2-fold—if at all. The 4 up-regulated genes are *Apol10b, Hbq1b, Fcho1*, and *D1gap5*. Among the 14 down-regulated genes, there are *Cd209b* and the 3 *Mups2, 17*, and *19*, which are known to encode pheromone-binding proteins. Other genes encode proteins which are involved in liver metabolism and these genes are down-regulated to about the same extent in vaccination-protected and non-vaccinated mice.

**Table 1:** **Genes, whose expression is up-or down-regulated more than 10-fold (*p* < 0.01) in the liver of both non-vaccinated (N) and vaccinated mice (V) infected with *P. chabaudi* on day 11 *p.i.* (Vd11, Nd11) in comparison to constitutive expression on day 0 *p.i.* (Vd0, Nd0)**.

**Gene**	**Gene description**	**RefSeq ID Agilent ID**	**Vd11 vs Vd0**.	***p*-value**	**Nd11 vs Nd0**.	***p*-value**	**Function (according to www.genecards.org)**
**UP-REGULATED GENES**							
*Apol10b*	Apolipoprotein L 10B	NM_177820 A_66_P120380	14	0.006	33	<0.001	“The protein encoded by this gene is a member of the apolipoprotein L family and may play a role in lipid exchange and transport throughout the body, as well as in reverse cholesterol transport from peripheral cells to the liver.”
*Dlgap5*	Discs, large (Drosophila) homolog-associated protein 5	NM_144553 A_55_P2050439	10	0.004	13	0.006	“Potential cell cycle regulator that may play a role in carcinogenesis of cancer cells. Mitotic phosphoprotein regulated by the ubiquitin-proteasome pathway. Key regulator of adherens junction integrity and differentiation that may be involved in CDH1-mediated adhesion and signaling in epithelial cells.”
*Fcho1*	FCH domain only 1	NM_028715 A_55_P2047461	12	0.004	10	0.007	“Functions in an early step of clathrin-mediated endocytosis. Has both a membrane binding/bending activity and the ability to recruit proteins essential to the formation of functional clathrin-coated pits. May regulate Bmp signaling by regulating clathrin-mediated endocytosis of Bmp receptors.”
*Hbq1b*	Hemoglobin, theta 1B	NM_001033981 A_52_P87503	13	0.008	23	0.005	“Theta-globin mRNA is found in human fetal erythroid tissue but not in adult erythroid or other non-erythroid tissue.”
**DOWN-REGULATED GENES**							
*Cd209b*	CD209b antigen	NM_001037800 A_52_P267717	0.09	0.006	0.06	0.002	“Pathogen-recognition receptor expressed on the surface of immature dendritic cells (DCs) and involved in initiation of primary immune response. Thought to mediate the endocytosis of pathogens which are subsequently degraded in lysosomal compartments. The receptor returns to the cell membrane surface and the pathogen-derived antigens are presented to resting T-cells via MHC class II proteins to initiate the adaptive immune response.”
*Cyp51*	Cytochrome P450, family 51	NM_020010 A_52_P164161	0.09	0.003	0.09	<0.001	Phase I metabolism.
*Cyp8b1*	Cytochrome P450, family 8, subfamily b, polypeptide 1	NM_010012 A_51_P266618	0.06	<0.001	0.07	0.009	“Involved in bile acid synthesis and is responsible for the conversion of 7 alpha-hydroxy-4-cholesten-3-one into 7 alpha, 12 alpha-dihydroxy-4-cholesten-3-one. Responsible for the balance between formation of cholic acid and chenodeoxycholic acid.”
*Hmgcs2*	3-hydroxy-3-methylglutaryl-Coenzyme A synthase 2	NM_008256 A_55_P1992582	0.09	0.007	0.01	<0.001	“This enzyme condenses acetyl-CoA with acetoacetyl-CoA to form HMG-CoA, which is the substrate for HMG-CoA reductase.”
*Idi1*	Isopentenyl-diphosphate delta isomerase	NM_145360 A_55_P2028961	0.08	0.001	0.04	<0.001	“IDI1 encodes a peroxisomally-localized enzyme that catalyzes the interconversion of isopentenyl diphosphate (IPP) to its highly electrophilic isomer, dimethylallyl diphosphate (DMAPP), which are the substrates for the successive reaction that results in the synthesis of farnesyl diphosphate and, ultimately, cholesterol.”
*Mup17*	Major urinary protein 17	NM_001200006 A_55_P2076196	0.02	0.007	<0.01	0.001	“Binds pheromones, likely to displace pheromones complexed to urinary MUPs and transport them to the vomeronasal organ (VNO) where they associate with their neuronal receptor(s).”
*Mup19*	Major urinary protein 19	NM_001135127 A_55_P1974080	0.03	<0.001	0.01	0.002	“Binds pheromones, likely to displace pheromones complexed to urinary MUPs and transport them to the vomeronasal organ (VNO) where they associate with their neuronal receptor(s).”
*Mup2*	Major urinary protein 2	NM_001045550 A_55_P2010097	<0.01	0.004	<0.01	0.005	“Binds pheromones, likely to displace pheromones complexed to urinary MUPs and transport them to the vomeronasal organ (VNO) where they associate with their neuronal receptor(s).”
*Ncmap*	Noncompact myelin associated protein	NM_001243306 A_55_P2148171	0.07	0.004	0.05	0.009	“Plays a role in myelin formation.”
*Prkcz*	Protein kinase C, zeta	NM_008860 A_52_P284889	0.09	<0.001	0.08	0.007	“Protein kinase C (PKC) zeta is a member of the PKC family of serine/threonine kinases which are involved in a variety of cellular processes such as proliferation, differentiation and secretion. Unlike the classical PKC isoenzymes which are calcium-dependent, PKC zeta exhibits a kinase activity which is independent of calcium and diacylglycerol but not of phosphatidylserine.”
*Sc4mol*	Sterol-C4-methyl oxidase-like	NM_025436 A_51_P209372	0.08	<0.001	0.08	<0.001	“The protein is localized to the endoplasmic reticulum membrane and is believed to function in cholesterol biosynthesis.”
*Serpina6*	Serine (or cysteine) peptidase inhibitor, clade A, member 6	NM_007618 A_51_P133562	0.08	0.006	<0.01	0.007	“This gene encodes an alpha-globulin protein with corticosteroid-binding properties. This is the major transport protein for glucorticoids and progestins in the blood of most vertebrates.”
*Slc22a7*	Solute carrier family 22 (organic anion transporter), member 7	NM_144856 A_51_P395856	0.04	0.001	<0.01	0.005	“Mediates sodium-independent multispecific organic anion transport. Transport of prostaglandin E2, prostaglandin F2, tetracycline, bumetanide, estrone sulfate, glutarate, dehydroepiandrosterone sulfate, allopurinol, 5-fluorouracil, paclitaxel, L-ascorbic acid, salicylate, ethotrexate, and alpha-ketoglutarate.”
*Slc2a5*	Solute carrier family 2 (facilitated glucose transporter), member 5	NM_019741 A_51_P514405	0.05	0.002	0.01	0.007	“Cytochalasin B-sensitive carrier. Seems to function primarily as a fructose transporter.”

Protective vaccination does not only induce self-healing infections of *P. chabaudi*, but also the number and identity of the malaria-induced genes in the liver at Vd11 differ from those identified in non-vaccinated mice at Nd11: 276 and 200 genes have been identified to be up- and down-regulated, respectively, more than 3-fold at Vd11 in relation to Vd0 (Figures 2A,B). Tables S7, S8 list the 43 and 112 genes genes whose expression levels are significantly (*p* < 0.01) up- and down-regulated between 3- and 10-fold at Vd11 in relation to Vd0 (Figures 2A,B). In the following, the focus of this study will be on those genes which are more than 10-fold (*p* < 0.01) up- or down-regulated by malaria in the liver of vaccination-protected mice at Vd11.

### Characterization of genes up-regulated in vaccination-protected mice

Among the 276 genes with up-regulated expression in the liver of only vaccination-protected mice toward the end of the crisis phase on day 11 *p.i.*, there are 60 genes, whose expression is more than 10-fold up-regulated at Vd11 in comparison to Vd0, respectively (Figure 2A). These 60 genes are listed in Table 2, which also provides information about the currently annotated functions of their encoded proteins. The 60 up-regulated genes can be subsumed under specific functional groups. The most prominent group contains genes, which can be even up-regulated by far more than 100-fold, and which code for different erythroid-associated proteins, in particular proteins associated with the surface membrane. For instance, the *Rhd* encodes the RhD blood group, *Add 2, Ank1*, and *Tmod1* encode proteins associated with membrane skeletal proteins, *Ermap* encodes a cell adhesion mediator, and *Slc4a1* an integral membrane protein. Besides these membrane-associated genes, this group of genes also contains *Gata1* and *Gfi1b* which are more than 70-fold up-regulated and which encode transcription factors involved in the development of erythroid cells. Another major group comprises genes which are associated with different phases of mitotis, such as *Aspm, Bub1b, Casc5, Ccna2, Cdca5, Cenpe, Diap3, Ercc6l, Hist1h2ab, Hist3h2ba, Kif4, Kif15, Kif18b, Mns1, Nusap1, Prc1, Sgol1, Ska1, Spag5, Ube2c*, and *Uhrf1*. Another group summarizes genes coding for proteins involved in cell cycle regulation, as e.g., *Birc5, Bub1, Ccnb1, Ccnb2, Cdc25b, Espl1, Melk, Mybl2, Plk1, Rgcc*, and *Rrm2*. The four genes *Bard1, Fanci, Neil3*, and *Top2a* code for proteins which have been described to be involved in DNA repair mechanisms. Moreover, the genes *Kcnn4a* and *Mki67*encode proteins involved in signaling and cell proliferation. Furthermore, the genes *Cd24a, Lrr1, Pbk, and Trim59* can be assigned to immunity. Two genes can be subgrouped to tissue permeability, among which is *Cldn13* that is more than 200-fold up-regulated. The encoded membrane protein claudin13 is known to be associated with tight junctions.

**Table 2:** **Genes expressed more than 10-fold (*p* < 0.01) in the liver of vaccinated mice infected with *P. chabaudi* on day 11 *p.i.* (Vd11) in comparison to constitutive expression on day 0 *p.i.* (Vd0)**.

**Gene**	**Gene description**	**RefSeq ID Agilent ID**	**Vd11 vs. Vd0**	***p*-value**	**Function (according to www.genecards.org)**
**ERYTHROPOIESIS**					
*Add2*	Adducin 2 (beta)	NM_001271859 A_55_P2063376	151	0.007	“Belongs to a family of membrane skeletal proteins involved in the assembly of spectrin-actin network in erythrocytes and at sites of cell-cell contact in epithelial tissues.”
*Ank1*	Ankyrin 1, erythroid	NM_001110783 A_55_P2063785	125	0.002	“Ankyrins are a family of proteins that link the integral membrane proteins to the underlying spectrin-actin cytoskeleton and play key roles in activities such as cell motility, activation, proliferation, contact and the maintenance of specialized membrane domains. Erythrocyte ankyrins also link spectrin to the cytoplasmic domain of the erythrocytes anion exchange protein.”
*Ermap*	Erythroblast membrane-associated protein	NM_013848 A_51_P391716	123	0.002	“The protein encoded by this gene is a cell surface transmembrane protein that may act as an erythroid cell receptor, possibly as a mediator of cell adhesion.”
*Gata1*	GATA binding protein 1	NM_008089 A_55_P2046852	113	0.003	“This gene encodes a protein which belongs to the GATA family of transcription factors. The protein plays an important role in erythroid development by regulating the switch of fetal hemoglobin to adult hemoglobin.”
*Gfi1b*	Growth factor independent 1B	NM_008114 A_51_P370678	79	0.003	“This gene encodes a zinc-finger containing transcriptional regulator that is primarily expressed in cells of hematopoietic lineage. Involved in control of expression of genes involved in the development and maturation of erythrocytes and megakaryocytes.”
*Rhd*	Rh blood group, D antigen	NM_011270 A_51_P241769	591	0.007	“The Rh blood group includes this gene, which encodes the RhD protein, and a second gene that encodes both the RhC and RhE antigens on a single polypeptide.”
*Slc4a1*	Solute carrier family 4 (anion exchanger), member 1	NM_011403 A_51_P196972	119	0.007	“The protein encoded by this gene is part of the anion exchanger (AE) family and is expressed in the erythrocyte plasma membrane, where it functions as a chloride/bicarbonate exchanger involved in carbon dioxide transport from tissues to lungs.”
*Tmod1*	Tropomodulin 1	NM_021883 A_51_P297069	15	<0.001	“The encoded protein is an actin-capping protein that regulates tropomyosin by binding to its N-terminus, inhibiting depolymerization and elongation of the pointed end of actin filaments and thereby influencing the structure of the erythrocyte membrane skeleton.”
**MITOSIS**					
*Aspm*	Asp (abnormal spindle)-like, microcephaly associated	NM_009791 A_55_P1988228	13	<0.001	“Probable role in mitotic spindle regulation and coordination of mitotic processes. May have a preferential role in regulating neurogenesis.”
*Bub1b*	Budding uninhibited by benzimidazoles 1 homolog, beta	NM_009773 A_51_P490509	16	0.005	“The protein has been localized to the kinetochore and plays a role in the inhibition of the anaphase-promoting complex/cyclosome (APC/C), delaying the onset of anaphase and ensuring proper chromosome segregation.”
*Casc5*	Cancer susceptibility candidate 5	NM_029617 A_51_P442964	13	0.004	“Performs two crucial functions during mitosis: it is essential for spindle-assembly checkpoint signaling and for correct chromosome alignment. Required for attachment of the kinetochores to the spindle microtubules.”
*Ccna2*	Cyclin A2	NM_009828 A_51_P481920	18	0.003	“Essential for the control of the cell cycle at the G1/S (start) and the G2/M (mitosis) transitions.”
*Cdca5*	Cell division cycle associated 5	NM_026410 A_51_P125135	78	0.005	“Regulator of sister chromatid cohesion in mitosis stabilizing cohesin complex association with chromatin. May antagonize the action of WAPAL which stimulates cohesin dissociation from chromatin.”
*Cenpe*	Centromere protein E	NM_173762 A_51_P164014	14	0.005	“Essential for the maintenance of chromosomal stability through efficient stabilization of microtubule capture at kinetochores. Plays a key role in the movement of chromosomes toward the metaphase plate during mitosis.”
*Diap3*	Diaphanous homolog 3	NM_019670 A_52_P104824	11	<0.001	“Binds to GTP-bound form of Rho and to profilin. Acts in a Rho-dependent manner to recruit profilin to the membrane, where it promotes actin polymerization. It is required for cytokinesis, stress fiber formation, and transcriptional activation of the serum response factor. DFR proteins couple Rho and Src tyrosine kinase during signaling and the regulation of actin dynamics.”
*Ercc6l*	Excision repair cross-complementing rodent repair deficiency complementation group 6 like	NM_146235 A_51_P123134	14	0.005	“DNA helicase that acts as an essential component of the spindle assembly checkpoint. Contributes to the mitotic checkpoint by recruiting MAD2 to kinetochores and monitoring tension on centromeric chromatin.”
*Hist1h2ab*	Histone cluster 1, H2ab	NM_175660 A_52_P420466	13	0.004	“Core component of nucleosome. Nucleosomes wrap and compact DNA into chromatin, limiting DNA accessibility to the cellular machineries which require DNA as a template.”
*Hist3h2ba*	Histone cluster 3, H2ba	NM_030082 A_55_P2036813	15	0.007	“Core component of nucleosome. Nucleosomes wrap and compact DNA into chromatin, limiting DNA accessibility to the cellular machineries which require DNA as a template.”
*Kif15*	Kinesin family member 15	NM_010620 A_52_P227391	15	<0.001	“Plus-end directed kinesin-like motor enzyme involved in mitotic spindle assembly Among its related pathways are Class I MHC mediated antigen processing and presentation and hemostasis.”
*Kif18b*	Kinesin family member 18B	NM_197959 A_52_P520466	24	<0.001	“In complex with KIF2C, constitutes the major microtubule plus-end depolymerizing activity in mitotic cells. Its major role may be to transport KIF2C and/or MAPRE1 along microtubules.”
*Kif4*	Kinesin family member 4	NM_008446 A_51_P254805	10	0.001	“Motor protein that translocates PRC1 to the plus ends of interdigitating spindle microtubules during the metaphase to anaphase transition, an essential step for the formation of an organized central spindle midzone and midbody and for successful cytokinesis. May play a role in mitotic chromosomal positioning and bipolar spindle stabilization.”
*Mns1*	Meiosis-specific nuclear structural protein 1	NM_008613 A_51_P100174	13	0.006	“May play a role in the control of meiotic division and germ cell differentiation through regulation of pairing and recombination during meiosis.”
*Nusap1*	Nucleolar and spindle associated protein 1	NM_133851 A_51_P240453	17	0.001	“NUSAP1 is a nucleolar-spindle-associated protein that plays a role in spindle microtubule organization.”
*Prc1*	Protein regulator of cytokinesis 1	NM_145150 A_55_P1988083	14	<0.001	“This gene encodes a protein that is involved in cytokinesis. The protein is present at high levels during the S and G2/M phases of mitosis but its levels drop dramatically when the cell exits mitosis and enters the G1 phase.”
*Sgol1*	Shugoshin-like 1	NM_028232 A_51_P487999	17	<0.001	“Plays a central role in chromosome cohesion during mitosis by preventing premature dissociation of cohesin complex from centromeres after prophase, when most of cohesin complex dissociates from chromosomes arms.”
*Ska1*	Spindle and kinetochore associated complex subunit 1	NM_025581 A_52_P139650	26	0.003	“Component of the SKA1 complex, a microtubule-binding subcomplex of the outer kinetochore that is essential for proper chromosome segregation. Required for timely anaphase onset during mitosis, when chromosomes undergo bipolar attachment on spindle microtubules leading to silencing of the spindle checkpoint.”
*Spag5*	Sperm associated antigen 5	NM_017407 A_51_P513530	19	<0.001	“Essential component of the mitotic spindle required for normal chromosome segregation and progression into anaphase.”
*Ube2c*	Ubiquitin-conjugating enzyme E2C	NM_026785 A_51_P451151	18	0.003	“Accepts ubiquitin from the E1 complex and catalyzes its covalent attachment to other proteins. Acts as an essential factor of the anaphase promoting complex/cyclosome, a cell cycle-regulated ubiquitin ligase that controls progression through mitosis.”
*Uhrf1*	Ubiquitin-like, containing PHD and RING finger domains, 1	NM_010931 A_55_P2035286	11	<0.001	“This gene encodes a member of a subfamily of RING-finger type E3 ubiquitin ligases. The protein binds to specific DNA sequences, and recruits a histone deacetylase to regulate gene expression. Its expression peaks at late G1 phase and continues during G2 and M phases of the cell cycle.”
**CELL CYCLE**					
*Birc5*	Baculoviral IAP repeat-containing 5	NM_001012273 A_55_P1983773	11	<0.001	“Multitasking protein that has dual roles in promoting cell proliferation and preventing apoptosis. Component of a chromosome passage protein complex (CPC) which is essential for chromosome alignment and segregation during mitosis and cytokinesis.”
*Bub1*	Budding uninhibited by benzimidazoles 1 homolog	NM_009772 A_51_P123405	15	0.006	“The encoded protein functions in part by phosphorylating members of the mitotic checkpoint complex and activating the spindle checkpoint. This protein also plays a role in inhibiting the activation of the anaphase promoting complex/cyclosome.”
*Ccnb1*	Cyclin B1	NM_172301 A_55_P2065671	14	0.009	“Essential for the control of the cell cycle at the G2/M (mitosis) transition.”
*Ccnb2*	Cyclin B2	NM_007630 A_51_P457528	12	<0.001	“Essential for the control of the cell cycle at the G2/M (mitosis) transition.”
*Cdc25b*	Cell division cycle 25B	NM_023117 A_52_P612382	12	0.005	“The protein is nuclear in the M and G1 phases of the cell cycle and moves to the cytoplasm during S and G2.”
*Espl1*	Extra spindle poles-like 1	NM_001014976 A_52_P658437	14	0.001	“Caspase-like protease, which plays a central role in the chromosome segregation by cleaving the SCC1/RAD21 subunit of the cohesin complex at the onset of anaphase.”
*Melk*	Maternal embryonic leucine zipper kinase	NM_010790 A_55_P2013336	15	<0.001	“Acts as a regulator of cell cycle, notably by mediating phosphorylation of CDC25B, promoting localization of CDC25B to the centrosome and the spindle poles during mitosis.”
*Mybl2*	Myeloblastosis oncogene-like 2	NM_008652 A_55_P1997141	16	0.003	“The protein encoded by this gene, a member of the MYB family of transcription factor genes, is a nuclear protein involved in cell cycle progression. The encoded protein is phosphorylated by cyclin A/cyclin-dependent kinase 2 during the S-phase of the cell cycle and possesses both activator and repressor activities.”
*Plk1*	Polo-like kinase 1	NM_011121 A_51_P344566	11	0.008	“Polo-like kinases (PLKs) are a family of four serine/threonine protein kinases that are critical regulators of cell cycle progression, mitosis, cytokinesis, and the DNA damage response.”
*Rgcc*	Regulator of cell cycle	NM_025427 A_51_P226269	14	0.003	“Modulates the activity of cell cycle-specific kinases. Enhances CDK1 activity. May contribute to the regulation of the cell cycle.”
*Rrm2*	Ribonucleotide reductase M2	NM_009104 A_55_P2173982	18	0.001	“Provides the precursors necessary for DNA synthesis. Catalyzes the biosynthesis of deoxyribonucleotides from the corresponding ribonucleotides. Inhibits Wnt signaling.”
**DNA REPAIR**					
*Bard1*	BRCA1 associated RING domain 1	NM_007525 A_55_P2186648	11	<0.001	“The BRCA1-BARD1 heterodimer specifically mediates the formation of Lys-6-linked polyubiquitin chains and coordinates a diverse range of cellular pathways such as DNA damage repair, ubiquitination and transcriptional regulation to maintain genomic stability. Plays a central role in the control of the cell cycle in response to DNA damage.”
*Fanci*	Fanconi anemia, complementation group I	NM_145946 A_55_P2025790	14	<0.001	“Plays an essential role in the repair of DNA double-strand breaks by homologous recombination and in the repair of interstrand DNA cross-links by promoting FANCD2 monoubiquitination by FANCL and participating in recruitment to DNA repair sites.”
*Neil3*	Nei like 3	NM_146208 A_52_P251366	35	0.003	“NEIL3 belongs to a class of DNA glycosylases homologous to the bacterial Fpg/Nei family. These glycosylases initiate the first step in base excision repair by cleaving bases damaged by reactive oxygen species and introducing a DNA strand break via the associated lyase reaction.”
*Top2a*	Topoisomerase (DNA) II alpha	NM_011623 A_55_P1995205	19	0.003	“Control of topological states of DNA by transient breakage and subsequent rejoining of DNA strands. Topoisomerase II makes double-strand breaks. Essential during mitosis and meiosis for proper segregation of daughter chromosomes.”
**CELL PROLIFERATION**					
*Kcnn4*	Potassium intermediate/small conductance calcium-activated channel, subfamily N, member 4	NM_008433 A_51_P389636	17	<0.001	“The protein encoded by this gene is part of a potentially heterotetrameric voltage-independent potassium channel that is activated by intracellular calcium. Activation is followed by membrane hyperpolarization, which promotes calcium influx. The encoded protein may be part of the predominant calcium-activated potassium channel in T-lymphocytes.”
*Mki67*	Antigen identified by monoclonal antibody Ki 67	NM_001081117 A_51_P253803	28	0.002	“This gene encodes a nuclear protein that is associated with and may be necessary for cellular proliferation.”
**IMMUNITY**					
*Cd24a*	CD24a antigen	NM_009846 A_52_P244193	33	0.005	“This gene encodes a sialoglycoprotein that is expressed on mature granulocytes and B cells and modulates growth and differentiation signals to these cells.”
*Lrr1*	Leucine rich repeat protein 1	NM_001081406 A_55_P2076941	12	0.003	“LRR1 specifically interacts with TNFRSF9/4-1BB, a member of the tumor necrosis factor receptor (TNFR) superfamily. Overexpression of this gene suppresses the activation of NF-kappa B induced by TNFRSF9 or TNF receptor-associated factor 2, which suggests that this protein is a negative regulator of TNFRSF9-mediated signaling cascades.”
*Pbk*	PDZ binding kinase	NM_023209 A_51_P230098	16	0.002	“This gene encodes a serine/threonine protein kinase related to the dual specific mitogen-activated protein kinase kinase (MAPKK) family. Evidence suggests that mitotic phosphorylation is required for its catalytic activity. The encoded protein may be involved in the activation of lymphoid cells.”
*Trim59*	Tripartite motif-containing 59	NM_025863 A_55_P2030938	16	<0.001	“May serve as a multifunctional regulator for innate immune signaling pathways.”
**TISSUE PERMEABILITY**					
*Cldn13*	Claudin 13	NM_020504 A_51_P469568	228	0.006	“Claudins are integral membrane proteins and components of tight junction strands. Tight junction strands serve as a physical barrier to prevent solutes and water from passing freely through the paracellular space between epithelial or endothelial cells.”
*Mfsd2b*	Major facilitator superfamily domain containing 2B	NM_001033488 A_55_P2159746	31	0.006	“Mfsd2a is expressed in blood vessels that form the blood–brain barrier (BBB). Knockout of Mfsd2a increases results in leaky BBB and in particular transcytosis without otherwise affecting tight-junctions. (PMID: 4828040)”
**MISCELLANEOUS**					
*Abcg4*	ATP-binding cassette, sub-family G (WHITE), member 4	NM_138955 A_55_P2018002	78	<0.001	“The protein encoded by this gene is included in the superfamily of ATP-binding cassette (ABC) transporters. This protein is a member of the White subfamily and is expressed predominantly in liver tissue.”
*Btnl10*	Butyrophilin-like 10	NM_138678 A_55_P2045931	68	0.002	Miscellaneous.
*Cd24a*	CD24a antigen	NM_009846 A_52_P244193	33	0.005	“This gene encodes a sialoglycoprotein that is expressed on mature granulocytes and B cells and modulates growth and differentiation signals to these cells.”
*Depdc1b*	DEP domain containing 1B	NM_178683 A_51_P303749	12	<0.001	Miscellaneous.
*Fam109b*	Family with sequence similarity 109, member B	NM_177391 A_55_P1972648	14	0.006	“Plays a role in endocytic trafficking. Required for receptor recycling from endosomes, both to the trans-Golgi network and the plasma membrane.”
*Fhdc1*	FH2 domain containing 1	NM_001205355 A_55_P1985693	43	<0.001	Miscellaneous.
*Nrip3*	Nuclear receptor interacting protein 3	NM_020610 A_55_P2000943	40	0.009	Miscellaneous.
*Prkar2b*	Protein kinase, cAMP dependent regulatory, type II beta	NM_011158 A_55_P2122200	15	<0.001	“Protein kinase A (PKA, aka cAMP-dependent protein kinase) is involved in the regulation of lipid and glucose metabolism and is a component of the signal transduction mechanism of certain GPCRs.”
*Retnlg*	Resistin like gamma	NM_181596 A_52_P425839	12	0.001	Miscellaneous.

**Figure 3:**
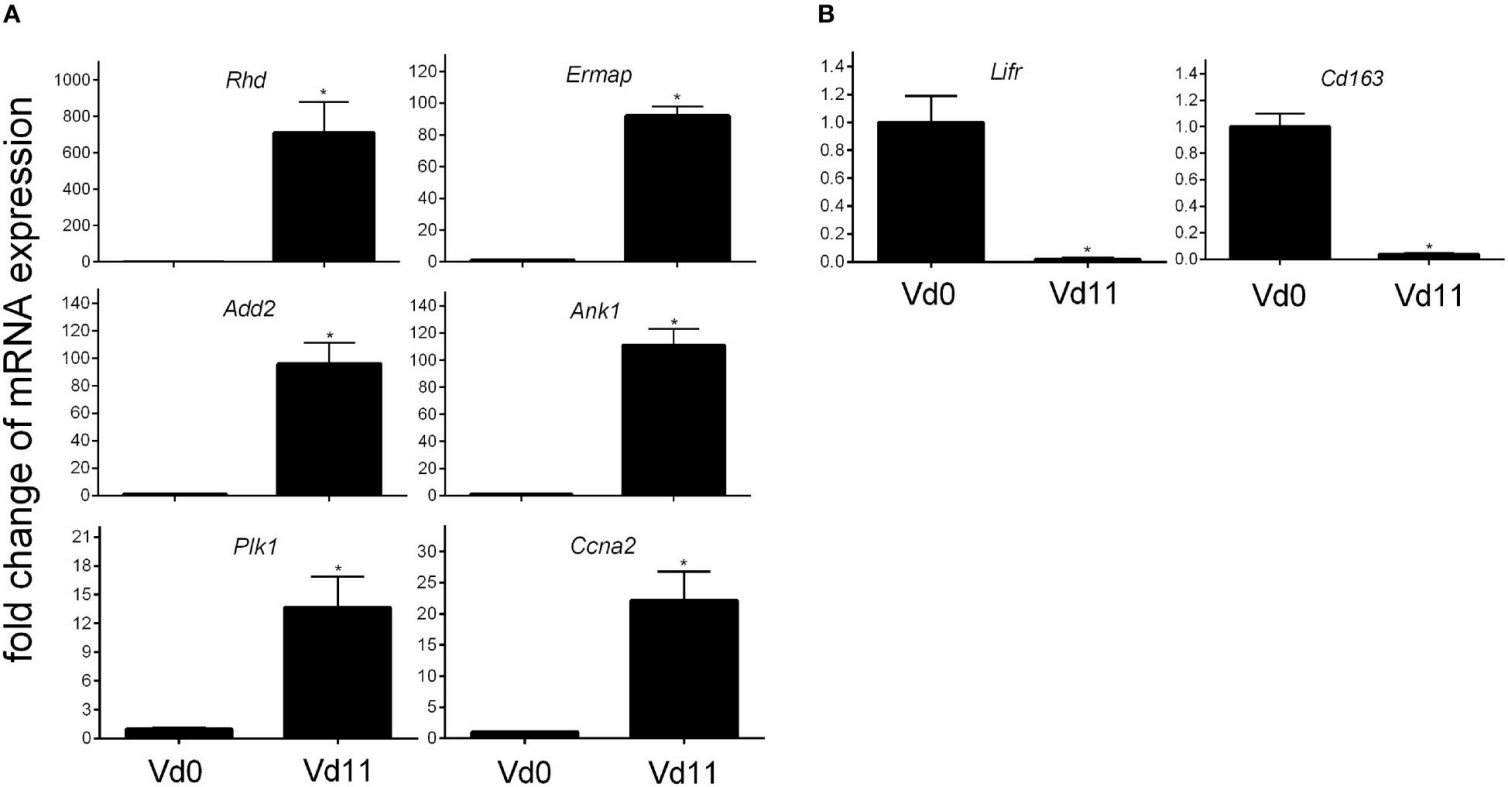
**Quantitative PCR of mRNAs of arbitrarily selected up-regulated (A) and down-regulated (B) genes in the liver of vaccinated Balb/c mice infected with *P. chabaudi* on day *0 p.i.* (Vd0) and on day 11 *p.i.* (Vd11)**. Values are means of triplicate determinations. Bars indicate half SEM and stars significant differences (*p* < 0.01) between Vd0 and Vd11.

Finally, quantitative PCR of arbitrarily selected up-regulated genes confirms that the expression of the erythroid-associated genes *Add2, Ank1, Ermap*, and *Rhd* are indeed up-regulated by more than 100-fold, while the genes *Ccna2* associated with mitosis and *Plk1* involved in cell cycle regulation are more than 10-fold expressed in the liver of vaccination-protected Balb/c mice infected with *P. chabaudi* on day 11 p.i. (Vd11) in relation to constitutive expression at Vd0 (Figure 3).

### Characterization of genes down-regulated in vaccination–protected mice

Among the 200 genes down-regulated in the liver of vaccination-protected mice at Vd11, only 5 genes in the liver respond to malaria with a significant down-regulation by more than 10-fold (*p* < 0.01) (Figure 2B). One of these 5 genes is *Cd163* which is down-regulated by approximately 50-fold (Table 3). The encoded CD163 is a transmembrane protein of 130 KDa expressed on Kupffer cells in the liver serving as a scavenger receptor cysteine-rich superfamily in the clearence and endocytosis of hemoglobin/haptoglobin complexes (Gronbaek et al., 2012; Etzerodt et al., 2013). Another gene is *Lifr*, whose expression is down-regulated by approximately 17-fold, encodes the transmembrane leukemia inhibitory factor (LIF) receptor (LIFR). Ectodomain shedding creates soluble receptor forms of both LIFR and CD163, which have different functions as the membrane-bound receptors (Layton et al., 1994; Owczarek et al., 1996; Moller, 2012; Ingels et al., 2013; Onishi and Zandstra, 2015). Quantitative PCR confirms that the expression of both *Lifr* and *Cd163* is strongly down-regulated at Vd11 as compared with Vd0 (Figure 3).

**Table 3:** **Genes down-regulated more than 10-fold (*p* < 0.01) in the liver of vaccinated mice infected with *P. chabaudi* on day 11 *p.i.* (Vd11) in comparison to constitutive expression on day 0 *p.i.* (Vd0)**.

**Gene**	**Gene description**	**RefSeq ID Agilent ID**	**Vd11 vs. Vd0**	***p*-value**	**Function (according to www.genecards.org)**
*Cd163*	CD163 antigen	NM_053094 A_55_P2289819	0.02	0.002	“The protein encoded by this gene is a member of the scavenger receptor cysteine-rich (SRCR) superfamily, and is exclusively expressed in monocytes and macrophages. It functions as an acute phase-regulated receptor involved in the clearance and endocytosis of hemoglobin/haptoglobin complexes by macrophages, and may thereby protect tissues from free hemoglobin-mediated oxidative damage. This protein may also function as an innate immune sensor for bacteria and inducer of local inflammation.”
*Dbp*	D site albumin promoter binding protein	NM_016974 A_55_P2032081	0.06	<0.001	“The protein encoded by this gene is a member of the PAR bZIP transcription factor family and binds to specific sequences in the promoters of several genes, such as albumin, CYP2A4, and CYP2A5.”
*Lifr*	Leukemia inhibitory factor receptor	NM_001113386 A_55_P2159264	0.06	<0.001	“Signal-transducing molecule. May have a common pathway with IL6ST. The soluble form inhibits the biological activity of LIF by blocking its binding to receptors on target cells.”
*Ncmap*	Noncompact myelin associated protein	NM_001243306 A_55_P2095039	0.08	0.005	“Plays a role in myelin formation.”
*Slc27a6*	Solute carrier family 27 (fatty acid transporter), member 6	NM_001081072 A_55_P2039379	0.09	0.005	“Involved in translocation of long-chain fatty acids (LFCA) across the plasma membrane.”

### Identification of differentially expressed lincRNAs

**Figure 4:**
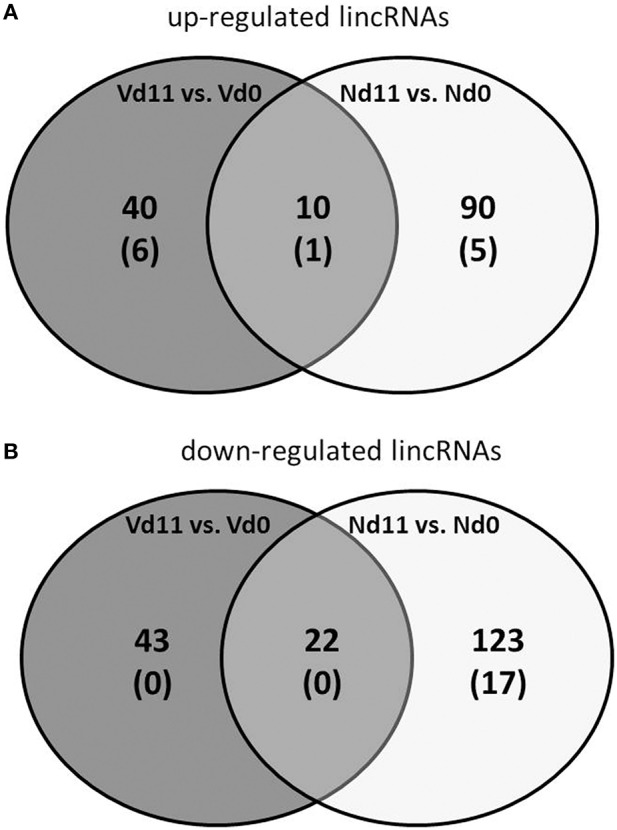
**Number of up-regulated (A) and down-regulated (B) lincRNAs expressed more than 3-fold (*p* < 0.01) in the liver of non-vaccinated (N) and vaccinated mice (V) infected with *P. chabaudi* on day 11 *p.i.* as compared to constitutive expressions on day 0 *p.i.* (Nd11 vs. Nd0 and Vd11 vs. Vd0)**. Numbers in brackets indicate lincRNAs expressed more than 10-fold.

Besides the 39,430 Entrez Gene RNAs, the used microarrays also contain 16,251 probes for detecting lincRNAs. In general, lincRNAs are known to range in length between 200 nts and about 100 kb, but lincRNAs are never transcribed into proteins. However, they are increasingly recognized as to play critical roles in different diseases, including diverse liver diseases (Takashi et al., 2014). We therefore also screened the microarrays for lincRNAs using the same stringent conditions as above for the mRNAs. Protective vaccinaton does not change the constitutive expression of lincRNA. However, malaria has induced changes in the expression of lincRNAs toward the end of the crisis phase, which differ between vaccination-protected mice and non-protected mice. The Venn-diagrams in Figure 4 show that, vaccination-protected mice, malaria induces 40 lincRNAs to be up-regulated more than 3-fold (Table S9), among which are 6 lincRNAs up-regulated by more than 10-fold at Vd11 in relation to Vd0. About the same number, namely 43 lincRNAs, are down-regulated by more than 3-fold in non-vaccinated mice at Vd11, but none of them is more than 10-fold down-regulated (Table S10). However, annotated functions are not yet available for any of the differentially regulated lincRNAs.

## Discussion

The vaccination procedure used here has been previously shown to protect female Balb/c mice from fatal *P. chabaudi* blood-stage malaria (Krücken et al., 2009). This becomes evident toward the end of the crisis phase on day 11 *p.i.*, when all non-vaccinated mice will succumb to infections, but the majority of the vaccinated mice will survive. This survival is associated with malaria-induced differential expression of genes and lincRNAs in the liver, as identified in the present study. These changes in gene expression are presumably associated with processes in the liver, which contribute to survival of blood-stage malaria of *P. chabaudi*.

Extramedullary erythropoiesis obviously is one of these malaria-activated processes in the liver of vaccination-protected mice. Indeed, we have detected a massive up-regulation of hepatic genes by far more than 100-fold, which encode diverse proteins of erythroid cells. In accordance, previous findings have provided evidence for the onset of hepatic erythropoiesis during blood-stage malaria in mice (Abo and Sekikawa, 2002; Halder et al., 2003; Wunderlich et al., 2014). It is therefore rather likely that reticulocytes produced in the liver contribute to the increasing number of peripheral reticulocytes in vaccination-induced self-healing infections of *P. chabaudi*. This increase begins at approximately peak parasitemia on day 8 *p.i.* and reaches its maximum of approximately 80% at the end of the crisis phase on approximately days 11 *p.i.*, when the number of peripheral erythrocytes has concomitantly declined to a minimum (Krücken et al., 2009). This dramatic decline of erythrocytes is presumably achieved both by mechanical destruction of *P. chabaudi*-infected erythrocytes, when the merozoite stages are released from erythrocytes, and by erythrophagocytosis mediated through natural auto-antibodies directed, for example, against clustered band 3 membrane proteins of senescent and aberrant erythrocytes including even *P. falciparum*-infected erythrocytes (Arese et al., 2005; Winograd et al., 2005; Lutz, 2012; Lutz and Bogdanova, 2013). In this context, it is worth mentioning that the spleen is rather likely not that organ which massively removes *P. chabaudi*-infected erythrocytes from circulation during the crisis phase of infection. Indeed, the spleen during crisis dramatically decreases the uptake of injected particulate material including fluorescently labeled *P. chabaudi*-infected erythrocytes (Krücken et al., 2005, 2009), while, concomitantly, the liver dramatically increases its capacity to trap injected particles. Thus, the liver, for example the Kupffer cells, rather than the “closed” spleen may massively remove *P. chabaudi*-infected erythrocytes from circulation during crisis, and this may even trigger hepatic erythropoiesis.

Anti-erythrocyte autoimmune mechanisms are obviously not directed against reticulocytes, since their number is increasingly replacing the declining number of erythrocytes during crisis. Moreover, the massive increase in reticulocytes is—at least transiently—advantageous for host survival, since reticulocytes, in contrast to erythrocytes, are not the preferred host cells of *P. chabaudi*. This non-preference may be due to the different composition and arrangement of plasma membrane-associated proteins of reticulocytes as compared with erythrocyte (Liu et al., 2010), which would make it more difficult for *P. chabaudi* to invade and/or survive in reticulocytes than in erythrocytes (Ott, 1968). This view is also supported by our finding showing an massive up-regulation of genes encoding erythroid skeletal membrane proteins such as *Add2* and *Ank1*. In this context, it is also noteworthy that C57BL/10 mice which have survived blood-stage malaria of *P. chabaudi* and subsequently acquired immunity to homologous re-infection have been previously shown to possess erythrocytes with a deficiency in the band 4.1a membrane protein (Wunderlich and Helwig, 1987). Erythrocytes deficient in 4.1a are known to be prone to ovalocytosis (Alloisio et al., 1983) and to be less penetrable by *Plasmodium* parasites (Hadley et al., 1983).

Furthermore, we have identified quite a number of up-regulated genes in the liver, which are known to control progress of cell cycle, mitosis and DNA repair. This may be, at least in part, also associated with increased hepatic erythropoiesis including enucleation of erythroblasts which may be regarded as an asymmetric form of mitosis (Thompson et al., 2010). However, the most straightforward interpretation of these data is that the liver of vaccination-protected mice, in comparison with non-vaccinated mice, undergoes accelerated regeneration toward the end of the crisis phase. This presumably enhances recovery of the liver from the pathological damages and injuries induced by the *P. chabaudi* infections. This view of accelerated liver regeneration may be also supported by our finding that the claudin13 encoding gene *Cldn13* is one of the most massively up-regulated genes in the liver of vaccination-protected mice toward the end of the crisis phase. In the mouse genome, *Cldn13* is one of 26 genes encoding claudins, which are 4-pass integral membrane proteins of 20–27 KDa and which are the main constituents of tight junctions (Morita et al., 1999; Lal-Nag and Morin, 2009). These restrict as a paracellular barrier the uncontrolled transfer of water and solutes in the intercellular space between cells of epithelia (Van Itallie and Anderson, 2004). The massive upregulation of *Cldn13* may therefore contribute to osmotic stability of liver tissue of the regenerating liver. However, there is also information available that the mouse-specific *Cldn13* encodes the most abundant claudin in the spleen of mice, where it has been predicted to be localized on the surface of erythroblasts and to be coordinately regulated as a part of stress-induced erythropoiesis by *Trypanosoma congolense* (Thompson et al., 2010). On the other hand, it is known that the liver is the only erythropoietic organ in the fetus, and the adult liver of mice is induced to resume erythropoiesis upon infection with the self-healing blood-stage malaria of *P. yoelii 17X* (Abo and Sekikawa, 2002; Halder et al., 2003). *De novo* clusters of erythroid cells have been shown in the parenchymal space of the liver. Thus, our finding, that *Cldn13* is massively upregulated in the liver of vaccinated Balb/c mice, may reflect a local expression of *Cldn13* in/around the microenvironment of erythroid clusters, thus protecting erythroid clusters and erythropoiesis, respectively, from osmotic perturbations. Local expressions of *Cldn13* have been also described for Sertoli cell tight junctions (Chakraborty et al., 2014) as well as for neonatal rabbit and rat proximal renal tubules (Abuazza et al., 2006).

Moreover, even down-regulated genes, for example *Lifr*, in the liver may contribute to enhanced liver regeneration in vaccination-protected mice. The LIF/LIFR system has been hypothesized to be involved in the expansion and differentiation of the liver stem cell compartment, which is activated during liver regeneration (Omori et al., 1996). Moreover, the LIF/LIFR system shares a common pathway through the common signal transducer GP130 with the polyfunctional interleukin 6 class cytokine family, consisting of IL-6, IL-11, OSM (oncostatin M), CNTF (cilary neurotrophic factor), CT-1 (cardiotrophin-1), and CLC (cardiotrophin-like cytokine) (Heinrich et al., 2003). These mutually interacting polyfunctional signaling networks are rather complex and involve both classic and trans-signaling mechanisms. For instance, IL-6 classic signaling takes place only in immune cells and hepatocytes expressing membrane-bound IL-6 receptor alpha. However, when bound to the soluble receptor created by ectodomain shedding from the membrane-bound receptor, IL-6 is then able to communicate by trans-signaling with all other cells through the ubiquitously expressed signal transducer GP130. IL-6 trans-signaling has been shown to play a decisive role in the regulation of liver regeneration (Scheller et al., 2011; Schmidt-Arras and Rose-John, 2016). Inhibition of IL-6 trans-signaling protects from *P. chabaudi* malaria (Wunderlich et al., 2012).

Moreover, even the differential expression of distinct lincRNA species with no annotated functions in the liver indicate that liver regeneration and hepatic erythropoiesis toward the end of the crisis phase require changes in the expression of diverse lincRNA species (Quagliata and Terraciano, 2014). Erythropoiesis has been recently shown to be associated with synthesis of diverse lncRNAs (Xu et al., 2013), targeted in part by the transcription factor GATA1 (Alvarez-Dominguez et al., 2014). The gene coding for GATA1 has been found here to be massively up-regulated by more than 100-fold in vaccination-protected mice toward the end of the crisis phase.

Collectively, our data indicate that protective vaccination against *P. chabaudi* malaria leads to changes in the liver, evidenced as altered gene expression in response to *P. chabaudi* malaria toward the end of the crisis phase. These changes in gene expression support the view that the dramatic decline of *P. chabaudi*-infected erythrocytes during crisis is counter-regulated by hepatic erythropoiesis leading to massive up-regulation of peripheral reticulocytes, which are not favored as host cells by *P. chabaudi*. Concomitantly, there is an accelerated regeneration of the liver. Both hepatic regeneration and erythropoiesis contribute to vaccination-induced survival of otherwise lethal blood-stage malaria of *P. chabaudi*.

## Author contributions

MD, SA, AA, and FW designed the study, MA and DD carried out the experiments and analyzed the data. All authors wrote and revised the manuscript.

### Conflict of interest statement

The authors declare that the research was conducted in the absence of any commercial or financial relationships that could be construed as a potential conflict of interest.
